# When Seeing Is Misleading: Clutter Leads to High-Confidence Errors

**DOI:** 10.1371/journal.pbio.0040077

**Published:** 2006-02-28

**Authors:** Liza Gross

Did you ever arrange to meet a friend at a busy street corner, then rush up to a total stranger thinking it was your friend? Neuroscientists have a theory to explain why such potentially embarrassing mistakes occur. We're trying to detect a target (our friend) amid a noisy background filled with distracters (a street filled with strangers) that impede our visual perception. Neuroscientists probe the underlying perceptual and neural processes of visual search by studying how distracters affect performance of a visual search task under controlled conditions in the lab.

Say a person is asked to pick out a target—a line tilted clockwise—that is embedded within a set of distracters—32 vertical lines. Signal detection theory (SDT) provides a framework for making quantitative predictions about the probability that an observer will detect a target under cluttered conditions. SDT assumes the brain represents each element in a visual search display as an independent variable with its own noise. It also assumes that when the observer isn't sure which stimulus is the target, she monitors all stimuli, and performance suffers. Thus, increasing the number of distracters (trying to find your friend on a busy street or a document on a messy desk) increases the background noise of the visual system's representation while reducing the accuracy and reaction time of performing the task.

One might intuitively expect that as noise and errors increase, confidence in one's decision plummets. But in a new study, Stefano Baldassi, Nicola Megna, and David Burr show that just the opposite happens. When observers searched for a tilted target embedded in distracters, they overestimated the magnitude of the tilt—and did so with a high degree of confidence in their decision.

It turns out that SDT lends a logical prediction to the seemingly counterintuitive finding that observers make more high-confidence errors when confronted with clutter. The prediction flows from a “squeaky wheel gets the grease” rule about visual processing, called the “Sign Max Rule.” When confronted with a set of independent, noisy responses, the visual system tends to base its decision on the largest response. Since each stimulus generates a noisy internal representation, and subjects monitor all the distracters to search for the target, as the number of distracters increases, the chance of perceiving a distracter as being more tilted than the target also increases. The authors predicted that the rule also applies to the confidence observers have in such “high magnitude” perceptual errors.

To test this prediction, the authors asked ten observers to indicate the direction and magnitude of the tilt of a target grating patch (small patches of blurred parallel black and white lines) tilted clockwise or counterclockwise. The target was briefly presented either alone or embedded in a circular array of vertical distracter patches. Though perceived magnitude is known to reflect observer confidence, the authors also got a direct measure by asking observers to report their level of confidence about each decision. Adding magnitude to the tilt discrimination task, the authors explain, helps shed light on the internal mechanisms that drive observers' decisions. As predicted by the Signed Max Model, estimates of perceived tilt increased with set size, as did the observers' confidence in their decisions. The authors conclude the visual system combines the outputs of noisy detectors and settles on the maximum signal.

These results suggest that the probability of being sure you saw something you didn't increases in chaotic environments, and could have far-reaching implications. The authors explain that while their study focused on “simple perceptual decisions about a single stimulus attribute,” the same type of processes may also apply to complex cognitive tasks involving problem solving and memory. If people find themselves confronted with multiple events in a chaotic, confusing environment, they may decide about some aspect of the situation and be totally wrong even though they have full confidence in their decision. The consequences of such a phenomenon could be relatively trivial, explaining why professional athletes often end up wasting their time arguing questionable calls with an official. Or they could prove a matter of life and death, perhaps accounting for why eyewitness testimony is so unreliable—or why soldiers sometimes can't tell friend from foe in the heat of battle.

## 

**Figure:**
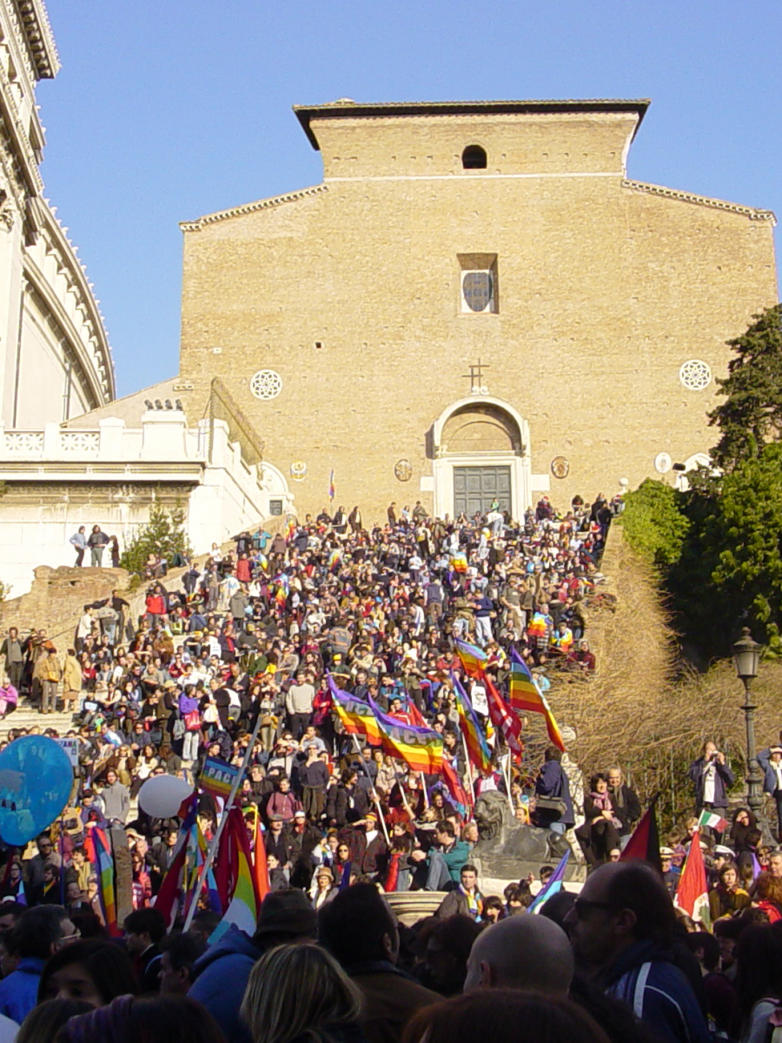
The probability of being sure you saw something you didn't increases in chaotic environments like this 2003 peace rally near the Campidoglio in Rome. (Image: Stefano Baldassi)

